# Exploring the Potential Applications of Wool Fibers in Composite Materials: A Review

**DOI:** 10.3390/polym16162360

**Published:** 2024-08-20

**Authors:** Alessia Patrucco, Marina Zoccola, Anastasia Anceschi

**Affiliations:** CNR-STIIMA, Institute of Intelligent Industrial Technologies and Systems for Advanced Manufacturing (STIIMA), Italian National Research Council (CNR), Corso G. Pella 16, 13900 Biella, Italy; alessia.patrucco@stiima.cnr.it (A.P.); marina.zoccola@stiima.cnr.it (M.Z.)

**Keywords:** wool waste valorization, composites, polymer composites, cement-based composites, sustainable materials

## Abstract

The use of renewable resources in composite materials is a vital strategy for enhancing sustainability in material science. Wool fibers are promising due to their unique properties, including thermal insulation and self-extinguishing characteristics. However, a substantial portion of wool is deemed unsuitable for textiles and is often discarded as waste. This review investigates the integration of wool fibers into polymer composites, aiming to improve sustainability and material performance. By analyzing recent advancements, this study highlights the potential of wool fibers to enhance the mechanical, thermal, and acoustic properties of composites. The findings support the development of eco-friendly materials that contribute to waste reduction and promote sustainable practices across various industries.

## 1. Introduction

**Figure 1:**
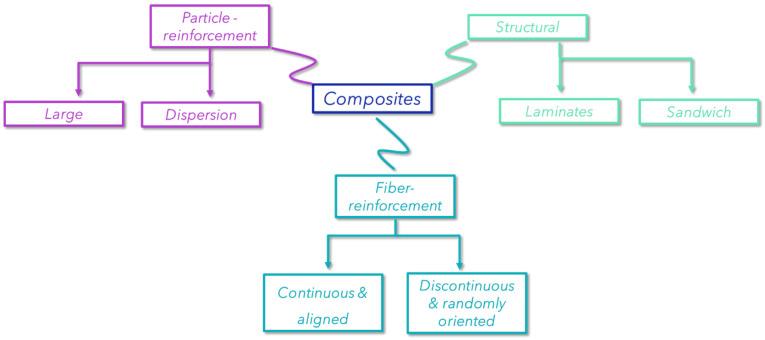
The most common types of composite materials.

Composite materials are a highly sought-after engineering product, defined as a heterogeneous material consisting of at least two different parts or elements. These materials are used in a wide variety of fields, including construction and advanced plastics. In general, composites exhibit superior properties compared to their mono-material counterparts, such as plastics, ceramics, or metals. They typically possess high strength and stiffness, combined with low density, and are lightweight. A variety of composite materials are discussed in the literature and a brief overview is given in Figure 1 [1].

**Figure 2:**
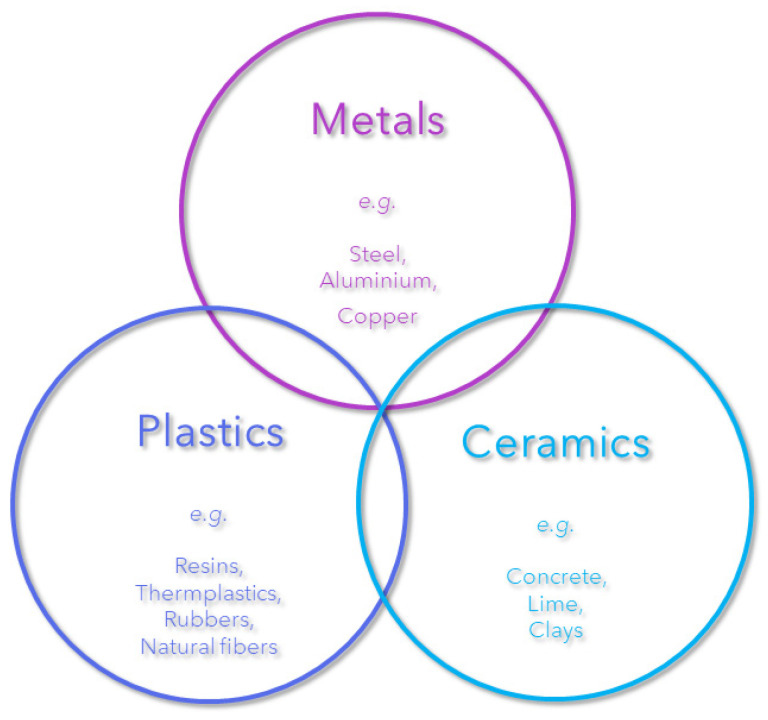
Relationship between the classes of materials used to make composites.

The present review will focus on a specific subset of composite materials, namely, those reinforced with fibers. Due to their small diameter compared to their length, fibers are able to increase the strength of the matrix, by forming a network, thereby improving the overall properties of the composite material. Moreover, fibers have a flexibility that allows them to be incorporated into complex manufacturing processes with relative ease. A variety of materials are used in the manufacture of composites, which are typically created by combining these materials and fall into the class of materials shown in Figure 2 [2].

Fibers used as reinforcement may be continuous and of considerable length, aligned with the direction of the loading, or they may be discontinuous. Continuous fiber composites are typically laminates, which are obtained by stacking single sheets of continuous fiber in a random orientation to achieve the desired stiffness and strength [1]. The use of continuous fiber in laminate form can have some drawbacks, for example when doubly curved shapes are required for manufacturing. To address this challenge, long fibers are typically woven into a fabric to achieve the desired orientation in the final product. In contrast, the use of short fibers appears promising due to their intrinsic ability to be arranged in composites in either unidirectional or multidirectional patterns, which enhances the strength and stiffness of the material. In addition, the use of short fibers circumvents the issue of curvature in manufacturing [2].

The majority of commercial fiber reinforcement is made from glass or carbon. However, with the pressing environmental concerns surrounding climate change and pollution, there is an urgent need to prioritize research of sustainable solutions and valuable alternatives. In response to the environmental challenges posed by climate change and pollution, the use of sustainable materials and processes has gained significant attention. Several materials have been explored and a noteworthy category includes natural fibers, which also have seen an increase in their presence on markets, particularly as a reinforcement material or filler [3]. However, three fundamental aspects need to be considered in composite materials: sustainability, performance, and cost. In this context, composite materials based on the combination of natural fibers and a carefully selected matrix may offer a viable solution for achieving a balance among these various aspects. Among the natural fibers, wool is emerging as a potential candidate for being incorporated into composite materials [4].

The term “wool” is often associated with textile and luxury applications, but not all wool can be applied for this purpose. Indeed, wool applied for textiles is almost fine wool, like merinos, but most EU wool is coarse wool that does not apply to the textile sector. The annual shearing of sheep, which is necessary for their well-being, produces 1.5–3 kg of coarse wool per animal, amounting to a total of over 200,000 tons in Europe. Coarse wool from EU sheep is essentially a worthless by-product, useless for textile applications. In addition, disposing of coarse wool in landfills is a significant cost for small-scale wool producers. To avoid these costs, many producers have illegally disposed of their waste by burying it with farm manure rather than sending it to a landfill. In addition, it is often burned, creating dangerous air and soil pollution [5].

In general, wool fibers possess a cylindrical shape and it has a cuticle composed chiefly of lipids, carbohydrates, and proteins, a cortex composed of about 90% keratin, and in coarse wool, there are some vacuoles known as medulla. Nevertheless, it is evident that the physical properties of wool fiber, including fineness and fiber length collectively dictate the end-use application. Consequently, in the subsequent section, a concise overview of some physical properties associated with wool is provided.

## 2. Physical and Chemical Properties of Wool Fibers

Wool fibers are usually classified mainly according to their fineness, and the resulting classification determines the applications for which they are suitable, as shown in Table 1.

A thorough comprehension of the physical and chemical properties of wool fiber can encourage researchers and industries to use wool fibers for a variety of purposes. The following section provides a brief overview of the diverse physical and chemical properties of wool fiber.

### 2.1. Physical Properties

The physical properties of wool are profoundly affected by its physical state, including diameter, degree of moisture absorption, number of scales, and the presence of ortho- and para-cortex cells. It is essential to consider these factors when developing composites.

### 2.2. Crimps

The term “crimp” is used to describe the waviness of wool fibers and covers a range of structures from fine, short ridges to larger, more substantial waves. The presence and distribution of crimps is a crucial aspect of wool quality, influencing the mechanical properties, spinnability, and luster of the final products. Typically, fine wool exhibits a higher number of crimps per inch, with merino wool having up to 30 crimps and coarse wool having one or two per inch.

### 2.3. Moisture Content

Wool is naturally hygroscopic because it is made up of protein, which has hydrophilic functional groups capable of absorbing water molecules. In addition, keratins are mostly organized in amorphous regions, which increase the hygroscopic properties of wool. Wool fiber has a capacity to absorb water, in the range of 13% to 50% relative to its weight, depending on the prevailing humidity conditions. The presence of absorbed water on wool fibers can affect the mechanical properties of the wool fibers [11].

### 2.4. Felting

The interlocking and contraction of wool fibers under the action of agents, such as heat, moisture, and friction are a property that has been exploited in the creation of a range of products, including hats, floor coverings, and soundproofing materials. This phenomenon, known as felting, can be attributed to the presence of surface scale, the microscopic structure that forms the wool fiber surface. In general, friction applied in the direction of the button to the top of the fiber will produce a smoother felting effect than that applied in the opposite direction. This property is known as *directional friction (DF)*, and can be calculated is possible using the Mercer equation (Equation (1)) [12].

(1) $$DF=\frac{\left(\mu_{2}-\mu_{1}\right)}{\left(\mu_{2}+\mu_{1}\right)}$$

where m_2_ is the coefficient of friction related to top-to-button contact while m_1_ is button-to-top contact.

### 2.5. Mecchanical Properties

Typically, wool has a high degree of elasticity, which allows it to stretch under load and return to its original shape when the load is removed. This elasticity can be attributed to both crimps and the proportion of ortho- and para-cortical cells within the fiber. The presence of a greater proportion of para-cortical cells appears to increase the elasticity of wool fibers [11]. Moreover, the presence of the alpha-helix conformation shows that keratins have a positive influence on the elastic recovery of wool fibers. Nevertheless, the presence of water on the surface and inside the fibers have an effect on the mechanical properties of wool. Indeed, wet wool loses approximately 25% of its strength and approximately 30% of its elasticity [9].

### 2.6. Chemical Properties

The chemical properties of wool are related to the presence of different functional or reactive groups. All reactions in which wool is involved typically originate in the side chain groups of accessible regions and subsequently continue in the cross-linking bond region, such as cysteine. Ultimately, these reactions culminate in the polypeptide bonds of the primary structure. It is essential that the chemical properties of wool fibers are carefully considered in the preparation of composite materials.

### 2.7. Effect of Acids

Wool, despite being a natural material, has a remarkable resistance to acidic conditions, which has led to the use of strong acids, such as sulfuric acid, to remove cellulosic-based residues in wool carbonizing. Under mild conditions, acids are capable to protonate basic functional groups, such as amines, making wool more reactive to anionic dyes and certain finishing agents [13]. However, the ability of wool to withstand acid exposure decrease with increasing temperature. Indeed, the use of hot acids can result in complete hydrolysis of wool.

### 2.8. Effect of Alkalis

Wool is highly susceptible to alkaline conditions, as the basic pH can readily disrupt the polypeptide bridge. However, when wool fibers are treated in a controlled and mild alkaline environment, the cysteine bonds can be transformed into hydrogen sulfide, which then forms new lanthionine bonds. This newly established linkage has a low level of reactivity, thereby rendering the wool fiber more stable. Furthermore, alkali treatment in the presence of heat affects the mechanical properties of wool, resulting in hydrolysis of aspartic and glutamic residues to aspartic acid and glutamic acid [9].

Having considered all the aspects relating to the wool fibers’ properties, the next section explores the role of wool fiber properties as reinforcement.

## 3. Wool Fibers-Based Composites

The manufacture of composites requires the use of two distinct elements: the matrix, which provides to provide the framework, and the reinforcement, which imparts various properties to the composite. The matrix also acts as the continuous component within the composite, facilitating the transfer of loads and giving the final shape to be manufactured. It also acts as a protective agent, protecting the reinforcing elements from mechanical and environmental deterioration. Various materials have been investigated as matrixes, including both inorganic and organic substances. In the field of wool-based composites, polymers are reported to be the most commonly used [14]. A variety of polymers can be used, including both thermoplastics and thermosets, although thermosets exhibit a broader range of applications.

**Figure 3:**
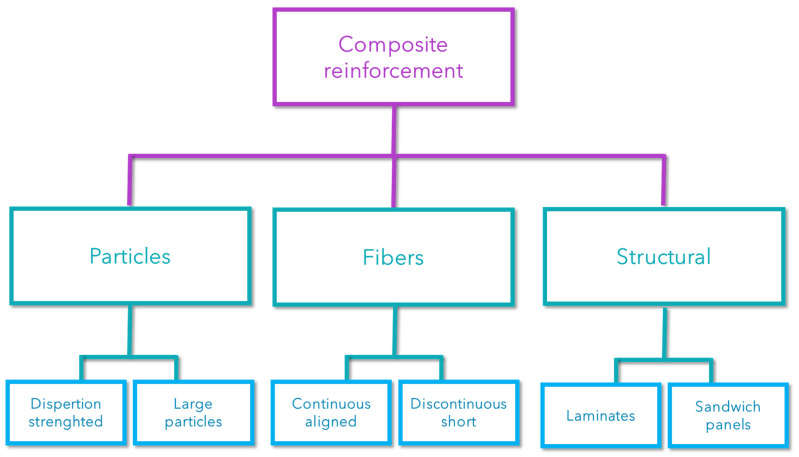
Scheme of different composites based on the shape of the reinforcement.

In terms of reinforcements, the materials used generally come in various forms. These materials can be classified according to their morphology, as shown in Figure 3.

Wool composites typically use wool in its natural form, as fibers. In fact, the weight of wool fibers is relatively low compared to other forms of reinforcement. In addition, wool fibers are inexpensive, biodegradable, fire resistant, and have excellent insulating properties. These characteristics make wool fibers an attractive reinforcement material. To confirm this hypothesis, J. Manivannan et al. carried out a comprehensive evaluation of sheep wool fibers to determine their suitability for various applications [15]. Using a range of characterization techniques, the researchers were able to investigate the properties of wool fibers in order to assess the feasibility of wool fibers as reinforcement. The results of this preliminary study on wool composites indicate the potential for the incorporation of wool fibers into composite materials. Indeed, wool fibers can be successfully incorporated into matrices of different nature. Wool fibers have been shown to be effective in polymer matrix, but also in mortar and concrete [16]. Sometimes the use of wool fiber is inhibited by some intrinsic characteristics of the matrix, such as pH in cement-based materials and affinity in polymer-based composites. Nevertheless, all these applications will be explored in this review and a brief overview of these innovative materials will be discussed in the next chapters.

### 3.1. Wool Fiber Reinforcement in Cement-Based Composites

**Figure 4:**
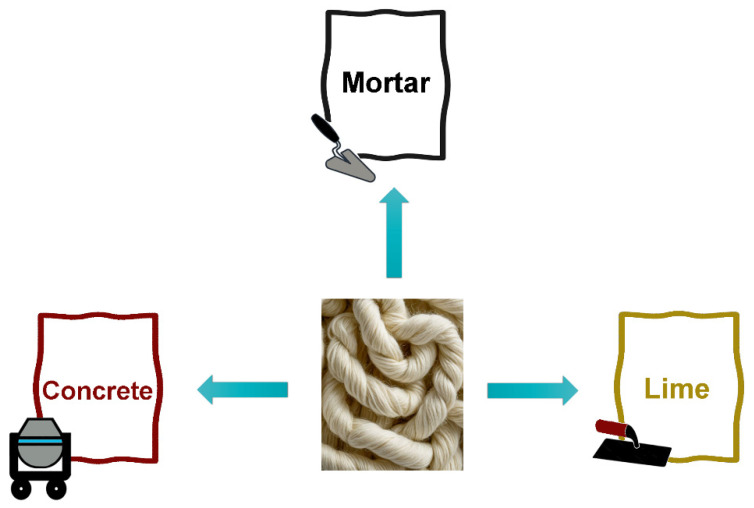
Wool fibers incorporated into different matrices.

Sheep wool is renowned for its intrinsic properties of thermal insulation, self-extinguishing properties, and acoustic insulation. In the past year, these properties have been extensively used in the building and construction industry. The first case of wool fiber being used as a reinforcing agent in cement-based composites dates back to 2010. In this study, researchers have investigated the potential of using clay, wool, and algae-derived compounds to produce wool-based bricks [17]. This work paves the way for the utilization of wool fibers in a variety of cement matrices, including mortar, lime, and concrete [18,19], as shown in Figure 4.

**Figure 5:**
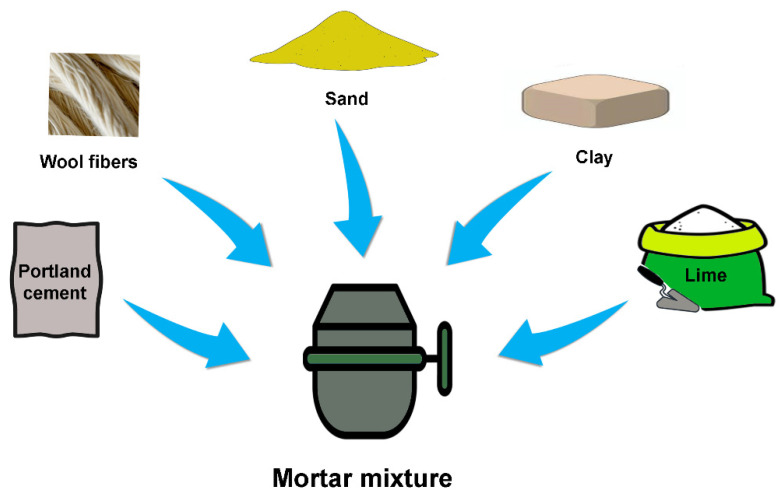
Mortar mixtures prepared with Portland cement, sand, lime, clay, and wool fibers.

As noted by Fantilli et al. [18], the pH of the cement matrix is a major challenge in the preparation of composites. It is, therefore, evident that cement-based materials typically have a high alkalinity, which can negatively affect the resistance of wool fibers. Nevertheless, the incorporation of wool fibers into concrete has shown promising results in terms of performance [20]. To enhance the effectiveness of wool fibers as reinforcements in concrete, Alyousef and colleagues proposed the incorporation of a high-range water-reduced admixture (HRWR) and a reduction in the number of dispersed wool fibers in the concrete mixture [21]. In contrast, Fantilli and coworkers demonstrated that reducing the alkalinity and humidity of the cement materials can markedly enhance the performance of the composites, increasing the capacity of the sheep wool fibers to bridge the crack surfaces and ensuring the retention of residual tensile strength [22]. In order to further mitigate the degradation of wool fibers in cement-based composites, two strategies can be employed. In the first scenario, the wool fiber may be subjected to a pre-treatment, whereas in the second case, modifications may be applied to the matrix. In other research endeavors, wool fibers were employed to enhance the mechanical and thermal properties of mortars and lime mortars. It was observed that the incorporation of wool fibers as reinforcement resulted in enhanced performance of the resulting composites [23]. In addition, the research conducted by Statuto et al. yielded intriguing results [24]. They found that the incorporation of 3% (*w*/*w*) sheep wool fibers and wheat straw as reinforcement into adobe clay led to notable improvements in the material’s mechanical properties. In this instance, the compression strength was found to be significantly enhanced, in comparison to the unmodified adobe clay [24]. Moreover, another study compared several key parameters, including compressive strength, flexural performance, thermal insulation, and density among different mortar mixes prepared with Portland cement, sand, lime, clay, and wool fibers [25,26], as shown in Figure 5.

The incorporation of wool fibers effectively reduced the density of the mortars, while also enhancing their thermal insulation properties. However, this resulted in a significant decrease in the mechanical performance of the composites. The mechanical properties of wool fibers and cement-based composites were deeply analyzed by Grădinaru et al. [27]. This study aimed to investigate the impact of sheep wool on the compressive strength of concrete. By testing various wool fiber and concrete mixtures, it was found that in some cases, the presence of wool can result in a reduction of approximately 15–30% in compressive strength compared to raw concrete. However, this reduction appears to be directly correlated with the length of the wool fibers. The optimum combination was found to be the use of wool fibers with a length of 55 ± 5 mm and the addition of no more than 0.35% (*w*/*w*) of wool fibers to concrete. This combination was shown to have a positive effect on the compressive strength. Conversely, some interesting results have been obtained using wool fibers as a reinforcing agent for mortar panels. For example, Cardinale et al. showed that optimizing the amount of wool fibers incorporated in the panels resulted in improved thermal and mechanical performance [28]. Similarly, Pederneiras et al. demonstrated that the incorporation of wool fibers into mortars can improve their flexural performance. The presence of fibers showed an increase in flexural strength of about 40% [23]. Other interesting results were obtained by Mobili and colleagues, who investigated the utilization of wool fibers as reinforcement for prefabricated clay sandwich panels for exterior enclosures [29]. It was observed that the incorporation of wool fibers increased the resistance to compression, bending, and shearing, while simultaneously reducing shrinkage. Furthermore, sheep wool plays another beneficial role in this context by absorbing moisture. The best results in terms of compressive strength, thermal insulation, and water absorption were observed by Erkmen et al. in their study on the incorporation of wool, kenaf, and wheat straw into concrete [30].

Other notable results have been achieved by incorporating wool fibers into cement-based composites used in the manufacture of insulation boards. It has been shown that the addition of wool fibers can result in a significant reduction in the mechanical behavior of insulation panels, but the insulation performance is significantly improved [26]. Furthermore, a study conducted by Fiore and co-workers demonstrated that varying the amount and length of wool fibers in cement mortars can effectively enhance the thermal insulation of the resulting products [31]. Nevertheless, the incorporation of wool fibers in cement-based composites has been shown to be an effective sound absorber. It has been shown that thin acoustic panels with incorporated wool fibers have a sound absorption coefficient comparable to that of rock wool and polyester [32]. Moreover, the findings indicated that the integration of wool fiber into panels is an effective approach for noise absorption, in alignment with the characteristics of other natural fibers, including wood wool and cellulose [16]. A comparison between the thermal and acoustic insulation properties of wool fiber-reinforced composites with common materials used for similar purposes is reported in Table 2.

Although wool fibers appear to enhance the mechanical properties of resulting composites and contribute to their sustainability, some drawbacks must be considered. These include the alkalinity of cement matrices and the natural biodegradation of wool fibers. For instance, Wei and Meyer examined the degradation pathway of natural fibers in alkaline and mineral-rich environments. The researchers discovered that the incorporation of metakaolin and the reduction of the alkaline environment of the matrix can effectively mitigate the deterioration of natural fibers [42]. Furthermore, Fantilli and co-workers demonstrated that the cement hydration process plays a pivotal role in the deterioration of natural fibers [22]. Indeed, the study revealed that the alkalinity of Portland cement exerts a significant influence on the stability of wool fibers, which in turn affects the mechanical properties of the final product. In particular, this research highlighted the influence of curing conditions, specifically the presence of water, on the degradation of wool fibers in composites. To address these drawbacks, some studies have concentrated their attention on the pre-treatment of wool fibers. For instance, the saltwater treatment modification on wool fibers has been demonstrated to be effective, resulting in an improvement in the mechanical properties and adhesion with the matrix [43]. Another effective methodology is based on the utilization of atmospheric plasma. The plasma treatment modifies the surface of the wool fiber, enhancing the surface interaction between cement-based materials and wool fibers. This results in the best mechanical and flexural performance of the composites [44,45].

Despite the ongoing limitations of research into wool fibers as a sustainable alternative to conventional reinforcing materials, the results to date appear promising. With some adjustments, wool fibers have the potential to effectively replace conventional building materials. Wool fibers are renewable and low-cost materials that can be used to further develop sustainable building materials. In addition, the incorporation of environmentally friendly components into composites will facilitate more sustainable dismantling and recycling of building materials at the end of their life.

#### Importance of Wool Fiber Size and Shape in Building Composites

The dimensions and shape of wool fibers have a considerable impact on the characteristics of composite materials, influencing parameters, such as mechanical strength, thermal insulation, and acoustic performance. It is crucial to comprehend these influences in order to achieve the most effective integration of wool fibers into building materials. In particular, the diameter of the fibers plays a pivotal role. The diameter of the fiber has a significant impact on the strength, insulation, and acoustic properties of the composite. Wool fibers with a diameter of 10 to 30 µm exhibit greater flexibility and are capable of forming a dense network within the composite, effectively enhancing its thermal insulation properties. Additionally, they improve the flexibility of the composite, which can reduce the probability of cracking under stress [31]. Moreover, finer fibers provide a higher surface area, which increases the ability of the composite to absorb sound, thereby improving its acoustic insulation. An additional crucial element, that may influence the ultimate characteristics of the composite, is the length of the fibers. The length of the fiber can impact the stability and workability of the manufacturing process. Indeed, longer fibers contribute to a more even load distribution within the composite, thereby enhancing its mechanical strength and stability [46]. They serve as reinforcement agents, improving the structural integrity of the material. While longer fibers impart strength, they may also impact the workability of the composite, necessitating meticulous attention during the mixing and application stages [47]. In addition to the influence of fiber diameter and length, the shape and degree of fiber crimp can also affect the properties of the resulting composite. Indeed, the natural crimp of wool fibers increases their ability to interlock within the composite matrix, thereby improving bonding and mechanical cohesion. This results in enhanced overall durability and resilience of the composite. However, the crimp and shape of the fibers can impact the porosity of the composite, which in turn affects both its thermal and acoustic insulation capabilities. It is of significant importance to ensure the appropriate control of fiber orientation and distribution in order to optimize the aforementioned properties. Additionally, the internal fiber structure can impact the final properties of the composites. Wool fibers are composed of a series of overlapping scales on their surface, known as the cuticle. The cortex, situated beneath the cuticle, is comprised of two distinct cell types that are capable of swelling and retaining moisture. Moreover, the core of the fiber, called the medulla, contains air-filled cavities. These hollow regions within the fiber contribute to the natural thermal insulation properties of wool by reducing its overall density and improving its ability to trap air [48].

The crimped and hollow structure of wool fibers is a crucial factor in the formation of air pockets within the composite material.

The presence of air pockets within the structure of the material serves to significantly enhance its thermal insulation properties by minimizing heat transfer. Indeed, the air is an ineffective conductor of heat, thus the trapped air pockets act as insulating barriers that impede the transfer of heat through the material. The natural crimp and structure of wool fibers provide a unique advantage for thermal insulation applications in building materials. By incorporating wool fibers into cement-based composites, manufacturers can develop materials that not only offer excellent thermal insulation but also improve energy efficiency and reduce environmental impact. This makes wool fiber composites an appealing choice for sustainable construction methodologies, notably in areas experiencing significant temperature fluctuations.

All these factors must be taken into account during the manufacturing process of the composites. The dimensions and configuration of wool fibers have a pivotal influence on the performance of composites, affecting mechanical strength, thermal and acoustic insulation, and overall durability. Through the strategic selection and processing of wool fibers, it is possible to create composite materials that are specifically tailored to meet the demands of building applications. This approach contributes to the development of sustainable and high-performance construction materials.

### 3.2. Wool Fiber Reinforcement in Polymer-Based Composites

Another significant area of potential exploitation for wool fiber is in polymer science. In light of the potential for developing novel bio-products to enhance the sustainability of plastic materials, the use of natural fiber as reinforcement has emerged as a promising field of research. In particular, wool fibers have been a primary focus among the diverse natural fibers because it is an abundant and renewable resource, offering a cost-effective alternative to other reinforcement materials, such as carbon fiber or glass fibers [49]. As mentioned above, wool fibers can impart a number of beneficial properties to the final products, including thermal and acoustic insulation properties, high mechanical properties, cost-effectiveness, biodegradability, and lightweight. These properties make wool fibers an ideal reinforcing material for polymer-based composites.

**Figure 6:**
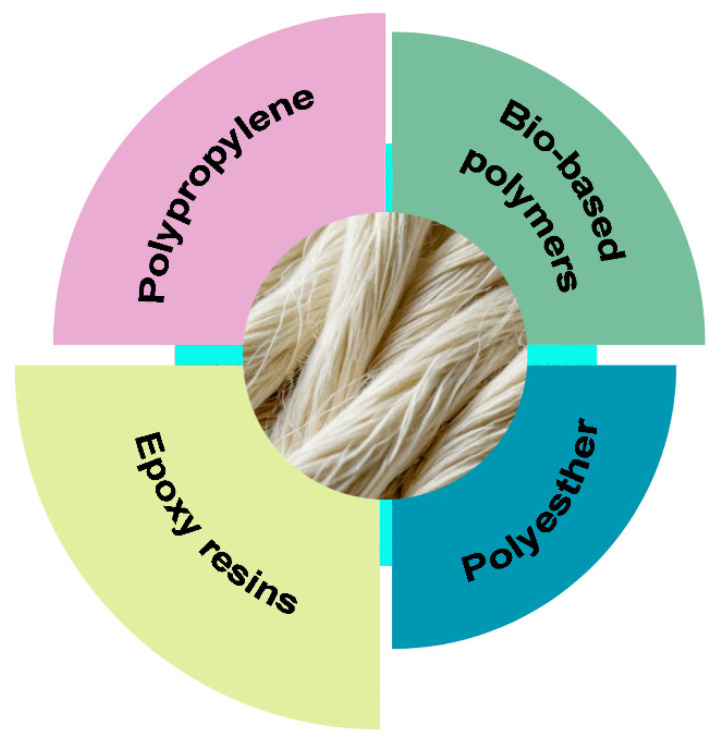
Wool fibers in different matrices.

Wool fibers can be successfully incorporated into a variety of polymer matrices, as shown in Figure 6.

Wool fiber composites manufactured from thermoplastic polymers are the most renowned. Indeed, thermoplastics products are utilized in a multitude of ways in everyday life and can range from simple objects to sophisticated applications. The principal factor enabling the success of these materials can be attributed to their versatility and low processing costs. Moreover, the properties of polymers can be modified through the use of various techniques, with the most common being the incorporation of different types of additives or fillers. It is therefore evident that the incorporation of wool fibers has resulted from a natural progression of events. Nevertheless, it is essential to recognize the potential limitations associated with the fabrication of wool fiber thermoplastic polymer composites. These drawbacks can be attributed to the morphological and chemical nature of the wool fibers, which affect the mechanical and thermal properties of the resulting product. The mechanical strength of the composites is directly proportional to the quantity of fibers incorporated. It is commonly observed that an increase in the quantity of wool fibers incorporated into the final product results in a reduction in the mechanical strength of the composite due to thermal attrition during the compounding process [50]. Moreover, the mechanical properties of polymer-based composites are also significantly influenced by the degree of compatibility between the hydrophobic nature of the thermoplastic matrix and the hydrophilic nature of the wool fibers. This incompatibility results in a lack of interfacial adhesion between the matrix and the wool fiber, which ultimately leads to a reduction in the mechanical properties of the resulting composites [33].

Notwithstanding these considerations, wool fibers have been investigated as a reinforcement in a variety of matrices. In a study conducted by Manivan and colleagues, sheep wool was applied as a reinforcement in a polyester matrix at varying weight ratios [51]. Unexpectedly, an increase in the amount of wool fibers resulted in a corresponding increase in tensile strength and hardness. The optimum ratio was found to be 50% w/w wool fibers in the composite. In addition, the incorporation of wool fibers into polyester composites has been identified as a promising avenue of research, as these composites exhibit favorable mechanical properties and impact resistance. Another significant research effort has been the incorporation of wool fibers into epoxy resins [52]. The results demonstrate that the incorporation of wool fibers into epoxy-based materials has no adverse effect on the mechanical properties of the materials, nor their shear properties. Nevertheless, through thermogravimetric analysis, the thermal insulation characteristics of the resulting composite were evaluated. It was found that the addition of the wool fiber caused a notable increase in thermal properties, accompanied by a reduction in the coefficient of thermal conductivity and an increase in the decomposition temperature. This indicates a shift towards a more thermally stable state, with an enhanced resistance to heat. Moreover, Strážnický et al. focused their studies on the effect of wool fibers on the properties of several thermosetting polymers [53]. In order to determine the effect of the presence of wool fibers in several thermosetting polymers, a specific sample set was employed as the basis for experimentation. The parameters considered included ductility, tensile strength, and deformation work. Following a comprehensive statistical analysis, the results demonstrated that the incorporation of 3% wool fibers into thermoset matrices is a viable approach for modifying the mechanical properties of this kind of composite. In a separate study, the physical and chemical properties of sheep wool incorporated into an epoxy resin based on bisphenol-A were investigated [54]. In this case, the incorporation of 50% wool fibers resulted in a notable increase in water adsorption, compared to the composite containing 40% wool fibers. A biodegradation test was also conducted, and the results indicated that both the concentration and the composition of the composite had a significant impact on the rate of biodegradation. Nevertheless, the incorporation of wool fibers appears to enhance the mechanical properties of these epoxy-based composites. The failure analysis of the composite made with epoxy resins and wool fibers as reinforcement has been explored by Barhat et al. In this study, the authors employed an open-hole tension test to assess the composite’s failure characteristics. A calculation was performed to determine that the tensile strength of the composite is dependent on the diameter of the hole by a factor of 0.09 [54]. Sharma and colleagues investigated the assessment of the mechanical and thermal characteristics of epoxy composites utilizing diverse sheep wool waste, encompassing both woven and non-woven materials [55]. By employing the vacuum-assisted resin transfer molding technique for the fabrication of the composite, the researchers were able to evaluate a range of parameters and properties inherent to the final product. The findings illustrate the potential of these composites to offer enhanced mechanical and thermal characteristics for diverse structural insulation applications, conferring numerous advantages including superior environmental performance, simplicity of use, minimal health effects, and energy-saving production.

**Figure 7:**
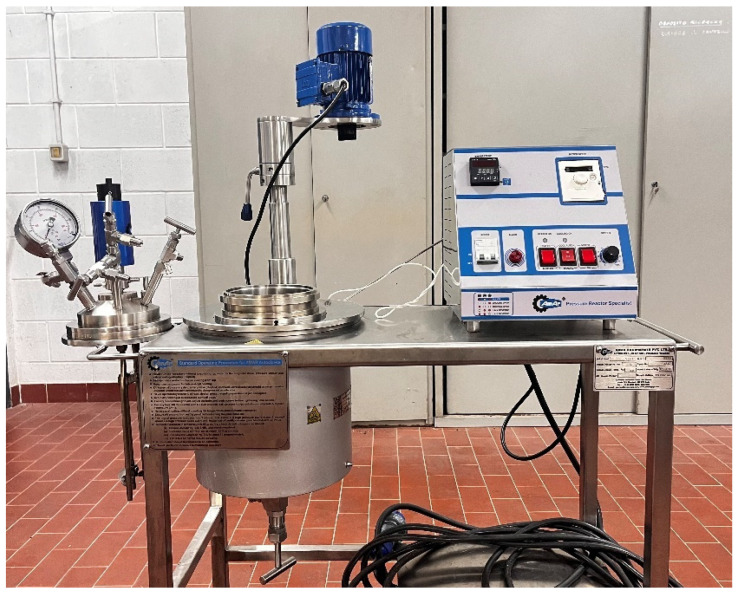
A lab-scale hydrolysis reactor.

Another interesting combination between wool fibers and a polymer matrix was carried out by Conzati et al. [56]. In this study, polypropylene-based composites were prepared by incorporating wool fibers at concentrations ranging from 20% to 60%. The isotropic composites were successfully prepared by the addition of a maleinized polypropylene compatibilizer, resulting in the fibers being dispersed effectively within the polymer matrix. The TGA analysis demonstrates the thermostability of the composites, emphasizing the synergistic interactions between the wool fibers and the matrix. In this instance, the incorporation of wool fibers appears to enhance the elastic modulus of the polypropylene-based composites, yet simultaneously impairs their tensile strength. It was postulated that this behavior was a consequence of the composite creation process. The blending phases were observed to result in the breakage of some fibers, which was identified as the probable cause of the reduction in fiber length. Better results in wool fiber-polypropylene-based composites have been obtained by Bertini and co-workers [57]. In this approach, in order to enhance the compatibility between the fibers and the polypropylene, the wool was subjected to a green hydrolysis process utilizing superheated water prior to the composite being formed. In this instance, the hydrolyzed wool fibers exhibit free amino acids, peptides, and low-molecular-weight proteins that could enhance compatibility with polypropylene. An example of a lab-scale hydrolysis reactor is reported in Figure 7.

Moreover, to enhance the adhesion between the polypropylene and wool fiber, maleic anhydride was grafted onto the surface of the polypropylene. The results indicated that the presence of hydrolyzed fibers has the potential to influence the thermo-oxidative degradation and crystallization of polypropylene, thereby enhancing the thermal resistance of the resulting composite. Furthermore, the incorporation of hydrolyzed wool fibers has enhanced the mechanical properties of the composite, exhibiting a gradual increase in elastic modulus with the addition of hydrolyzed fibers. In the field of polypropylene-based composite reinforced with wool, Salama et al. have presented an intriguing study [58]. In this work, the wool fibers were ground in a freeze mill in order to obtain a fine wool powder. The wool powder was incorporated into a polypropylene matrix using a twin-screw extruder to produce a laboratory-scale composite. Scanning electron microscopy revealed that the wool powder was homogenously distributed in the polymer matrix, while thermal and tensile properties demonstrated that the wool powder could be effectively applied in the production of composites.

In addition to polypropylene, the use of wool fibers in a polyester matrix has also been the subject of extensive study. To illustrate, Manivannan and colleagues have fabricated a series of randomly oriented composites utilizing three distinct weight percentages of wool fibers (ranging from 20% to 40%) via the compression molding technique [51]. The results of their experiments showed that the highest hardness can be achieved when 20% wool fibers are incorporated into the polyester matrix. Furthermore, the optimum tensile strength can be achieved by incorporating 40% wool fibers into the polymer matrix. Similar results were obtained by Conzatti and co-workers using a bio-based polyester [59]. They were able to successfully incorporate over 40% of wool fibers into the polymer matrix by using PVA as a compatibilizer. The adhesion between the fibers and the matrix was significantly improved, especially in the case of the PVA-treated fibers. This resulted in an increase in Young’s modulus of up to 500% compared to the pure polyester matrix. Conversely, as the majority of the wool fibers in the composites were shorter than the critical length, the effective reinforcing effect of the fibers on the matrix was limited, as evidenced by the reduced strength at break values. They were able to successfully incorporate over 40% of wool fiber into the polymer matrix through the use of PVA as a compatibilizer. The adhesion between the fibers and the matrix was notably enhanced, particularly in the case of PVA-treated fibers. This resulted in an increase in Young’s modulus by up to 500% in comparison to the neat polyester matrix. Conversely, given that the majority of wool fibers within the composites were shorter than the critical length, the effective reinforcing action of the fibers on the matrix was constrained, as evidenced by the diminished strength at break values.

Other noteworthy findings were reported by Pawlak et al. [60]. In this study, sheep wool fibers were functionalized using silanes and incorporated into a maleinized linseed oil and polylactic acid matrix. The results demonstrated that the silane functionalization significantly improved the interaction between the wool fiber surface and the matrix. With regard to the mechanical properties, an increase in the quantity of treated wool fibers has been observed to result in an enhancement of Young’s modulus and elongation at break. Furthermore, the incorporation of wool fibers into the composite material results in an increase in its hydrophobicity.

Another interesting research article on the use of wool fibers as reinforcements examines the development of sustainable bio-composites, with a particular focus on their potential applications in the packaging and agricultural sectors [61]. It is noteworthy that in this paper, wool waste has been applied and treated using a hydrolysis process in superheated water, with the intention of utilizing it in bio-composites in conjunction with kraft paper. The synthesis of 100% organic, compostable, and biodegradable kraft pulp/hydrolyzed wool bio-composites has yielded materials with satisfactory mechanical and insulating properties.

An additional interesting use of wool fiber as a composite reinforcement in the biomedical field is worthy of note. For example, Mangat and colleagues developed a porous scaffold with exceptional antibacterial properties [62]. In this study, wool fibers were treated in order to be successfully incorporated into a polylactic acid-based three-dimensional structure, and good mechanical resistance was also observed. Kelly et al. have developed a new composite material at the nanoscale level by combining wool fibers with silver nanoparticles [63]. In this approach, silver nanoparticles were bonded to wool fibers using trisodium citrate as a linker. The silver nanoparticle-wool composites display notable antimicrobial and antistatic characteristics. A comprehensive chemical and physical characterization, supported by a range of techniques, demonstrated the potential for bio-medical applications.

## 4. Importance of Wool Fiber-Matrix Adhesion in Composites

**Figure 8:**
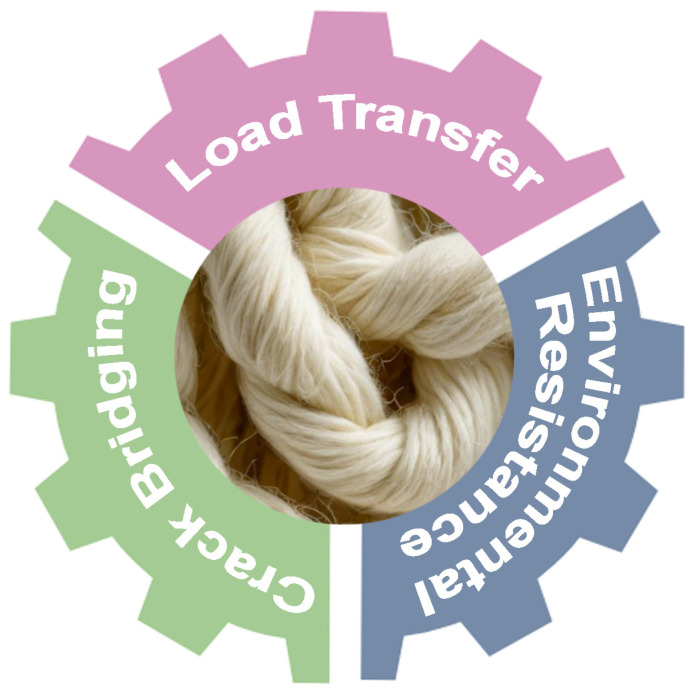
Important aspects of fiber-matrix adhesion.

Great attention is paid to the importance of fiber-matrix adhesion. In fiber-reinforced composites, the adhesion between the fibers and the matrix is of significant importance for the effective transfer of stress, the assurance of mechanical integrity, and the optimization of the composite performance [64]. The presence of a strong fiber-matrix adhesion allows for the transfer of loads in a more efficient manner, reduces the likelihood of delamination, and enhances the overall durability of the composite material [65]. In particular, effective adhesion enables the fibers to bear loads transferred from the matrix, thereby enhancing the strength and stiffness of the composite. This is of particular importance in the context of building materials, where structural integrity is of critical importance. Furthermore, robust adhesion enables fibers to bridge cracks in the matrix, increasing resistance to crack propagation and improving the toughness of the material [66]. In the case of the use of degradable and moisture-sensitive reinforcements, such as wool fiber, a favorable adhesion between the reinforcement and matrix can prevent the ingress of water and other agents that might degrade the composite over time [67]. All these aspects are summarized in Figure 8.

The adhesion between wool fibers and the matrix in building composites is influenced by a number of factors. The surface state of the fibers is of great importance. The surface topography and chemical composition of wool fibers can exert a considerable influence on their adhesion to the matrix. Wool fibers are coated with cuticle scales that can facilitate mechanical interlocking with the matrix. Indeed, the adhesion between wool fibers and the matrix can be enhanced through the application of surface treatments, such as alkali or silane treatments [60]. These treatments modify the fiber surface, increasing surface energy and introducing functional groups that interact with the matrix, consequently improving the bonding between the two components. Moreover, modifications can be made to the matrix formulation. It is of great importance to select an appropriate matrix for the fibers. However, the incorporation of compatibilizers or additives in the matrix can enhance adhesion by promoting chemical interactions between the fibers and the matrix [68]. Another significant factor that can prevent the optimal fiber-matrix adhesion is the orientation and distribution of the reinforcement [69]. The alignment and even distribution of fibers within the matrix can impact the strength of the adhesion. The orientation of fibers is a crucial factor in ensuring uniform stress distribution and effective load transfer across a composite material.

## 5. Preparation Techniques of Wool Fiber Composites

In the following paragraphs, the potential techniques for the creation of wool-reinforced composites will be explored.

### 5.1. Hand-Lay-Up

**Figure 9:**
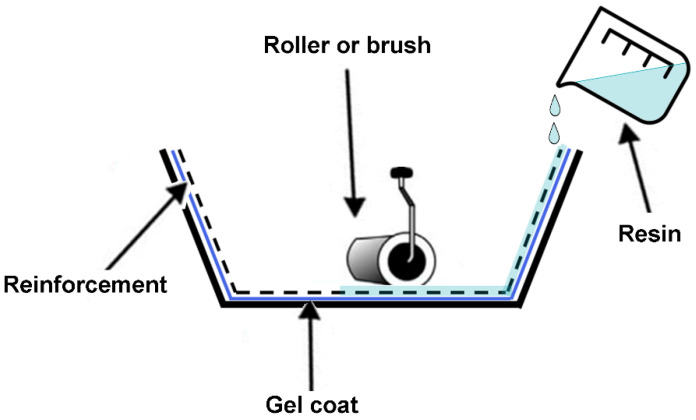
Hand-lay-up scheme.

In the hand-lay-up technique, the fibers are simply positioned within the mold in the appropriate orientation. Then the resin material is poured into the mold. Subsequently, a roller or brush is employed to moisten the fibers with resin and expel any air that may have become entrapped within the lay-ups, as shown in Figure 9. In certain instances, the addition of an antiadhesive agent can facilitate the subsequent removal of the composite from the mold [70].

### 5.2. Spray-Up

**Figure 10:**
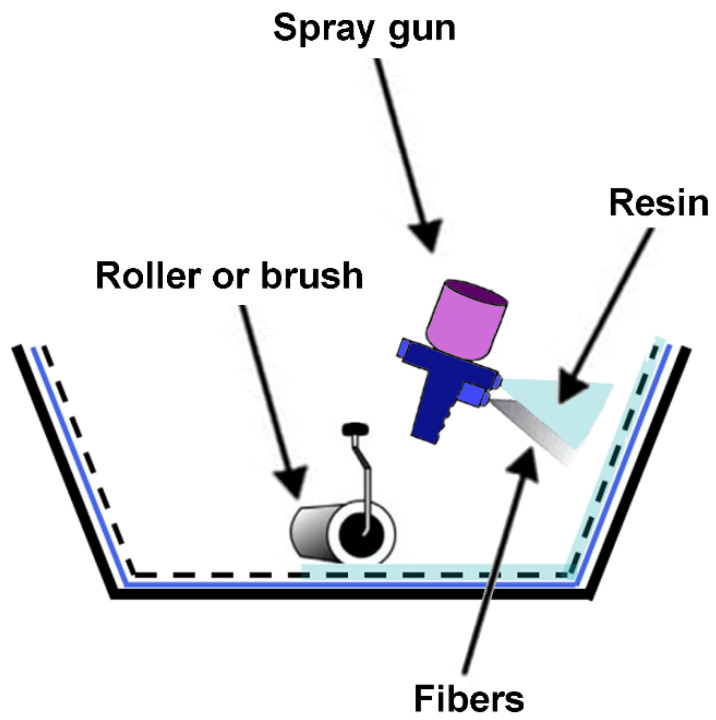
Spray-up schema.

The spray-up technique is a modification of the hand-lay-up method. In this case, fibers and resin are sprayed into the mold, and then a roller is used to moisten the fibers. This technique is faster than the hand-lay-up but the resulting manufacture is less homogenous (Figure 10) [71].

### 5.3. Resin Transfer Molding (RTM)

**Figure 11:**
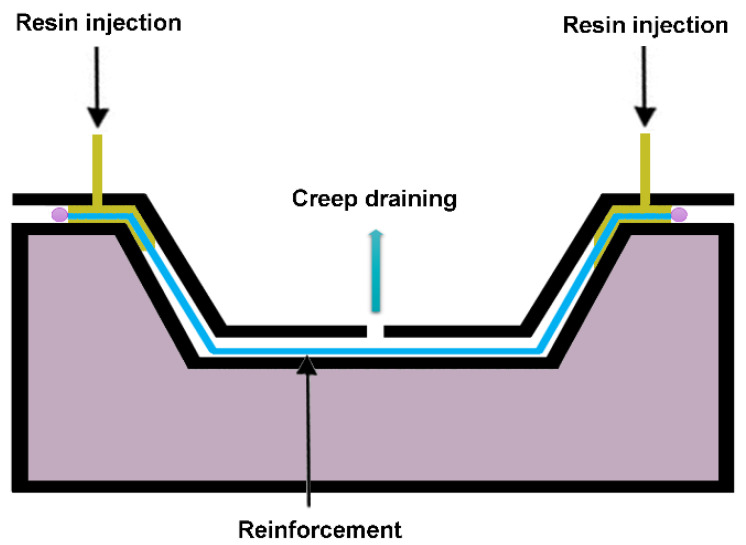
RTM scheme.

The resin transfer molding (RTM) process enables the production of composites that are dimensionally precise and possess a superior surface quality. In the RTM method, the fibers are inserted into the mold and then closed. Sometimes a preform tool can be used to stabilize the position of the fiber. A low-viscosity resin is then pumped into the mold, where it undergoes a curing process, as shown in Figure 11. This technique allows the production of composites with remarkable thickness [72].

### 5.4. Vacuum-Assisted Resin Transfer Molding (VaRTM)

**Figure 12:**
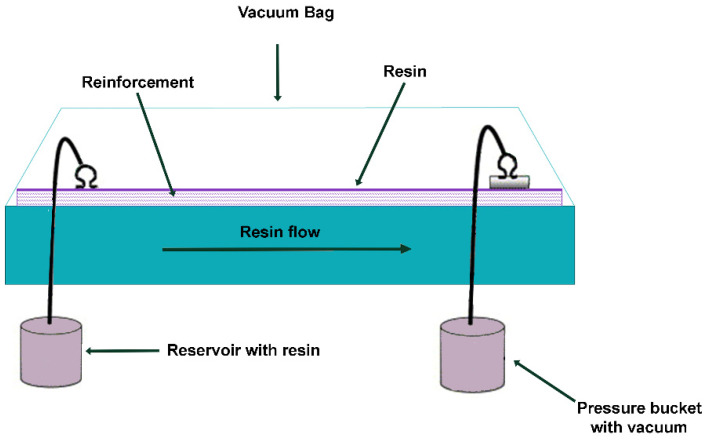
Vacuum-assisted resin transfer molding schema.

Vacuum-assisted resin transfer molding (VaRTM) or vacuum-injected molding (VIM) is a closed mold composite manufacturing methodology, as shown in Figure 12. In the vacuum bag technique employed in RTM, a vacuum is applied to the top portion of the mold tool, facilitating the formation of the composite material at a much higher rate than is possible with conventional techniques [73].

### 5.5. Resin Film Infusion (RFI)

**Figure 13:**
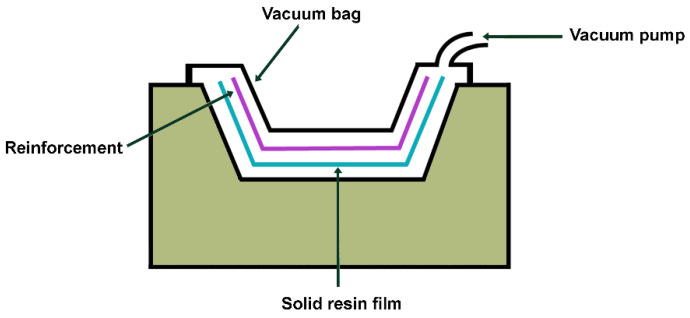
RFI scheme.

The resin film infusion (RFI) process is well-suited for the fabrication of relatively large structures and a summary of the process is presented in Figure 13. The process employs an open mold upon which successive layers of fibers and solid resin film are deposited. The materials are then hermetically sealed in a vacuum bag and the air is evacuated from the bag using a vacuum pump. The ensemble is then placed in an autoclave and subjected to a combination of pressure and heat. The temperature causes a reduction in viscosity, allowing the resin to flow into the fiber layers under pressure. Once the infusion process is complete, the resin is allowed to cure and solidify [74].

### 5.6. Compression Molding

**Figure 14:**
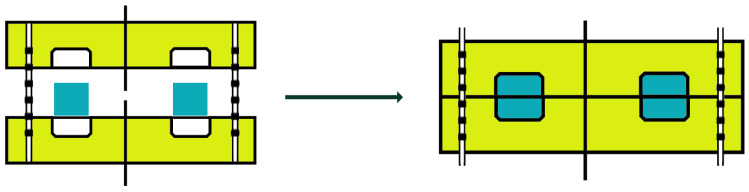
Compression molding scheme.

Compression molding is a process in which the material is typically preheated and then placed in an open and heated mold. The mold is then closed, and pressure is applied to force the material into perfect contact with the mold surfaces, as shown in Figure 14. In the case of a thermoplastic compound, the entire assembled component must be cooled under pressure before the composite is ejected from the mold. Conversely, forced cooling is not a necessary step for thermoset compounds [75].

### 5.7. Injection Molding

Injection molding is the most widely used manufacturing process for polymers and polymer composites, allowing the production of high-precision parts with complex geometries at a very low cost. The process consists of the following cyclic steps [76]:

(a) Loading the cylinder;
(b) Mold closing;
(c) Polymer plasticization;
(d) Injection/pressure;
(e) Cooling;
(f) Composite removal.

**Figure 15:**
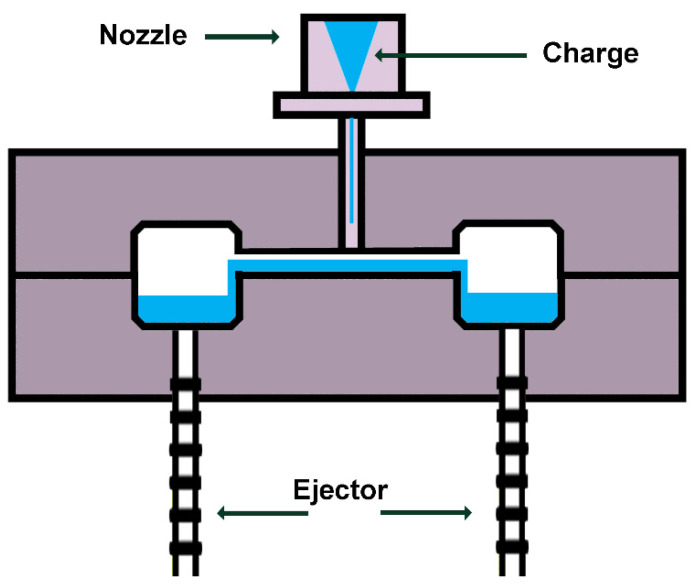
Injection molding schema.

The materials required to make the composites are introduced into a heated chamber, mixed by a helical-shaped screw, and then introduced into a mold cavity. The material is then subjected to a cooling and solidification process to achieve the desired shape. A schematic diagram is shown in Figure 15.

## 6. Application of Wool Fiber Reinforced Composites

**Figure 16:**
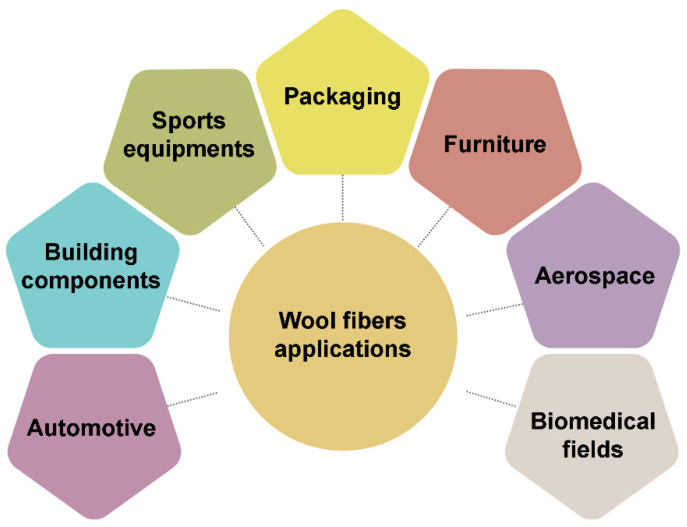
Wool fiber application in different sectors.

While wool has a wide range of applications in the textile industry, the majority of sheep breeds native to Europe are not suitable for textile production, and most are bred for meat production. In such cases, wool is considered a by-product and is usually disposed of in landfills. It is therefore necessary to develop strategies for the valorization of wool and to consider novel alternatives. The use of wool fibers as reinforcements in composites is an optimal way to valorize waste and reduce the amount of waste going to landfills. The use of wool as a filler in composite materials has the potential to be successfully applied in a wide range of sectors. For example, in the construction industry, the use of composite materials containing sheep wool fibers has been identified as a potential solution for insulation boards, wall panels, roofing materials, and a range of other building components. These materials offer a combination of lightness, durability, and excellent thermal insulation properties. In addition, these materials can be formulated to be fire resistant, which would enhance safety in building applications. In addition to their suitability for use in construction, these composites are also suitable for the manufacture of automotive and transport components, such as dashboards, interior trim, and door panels. This versatility and potential for widespread adoption is further illustrated in Figure 16 [49]:

Wool fibers can be successfully used in a wide variety of applications. For instance, wool fibers can be used to produce lightweight and durable sports equipment or to create eco-friendly materials for packaging [12,18]. Furthermore, wool-reinforced composites are employed in furniture production, particularly in the fabrication of chairs and tabletops. Additionally, they are utilized in the biomedical field for the production of orthopedic implants. Finally, wool fiber composites are utilized in the aerospace industry, particularly in aircraft interiors and structural components [49].

## 7. Limitations, Challenges, and Future Perspectives Related to the Incorporation of Wool Fibers into Composite Materials

**Figure 17:**
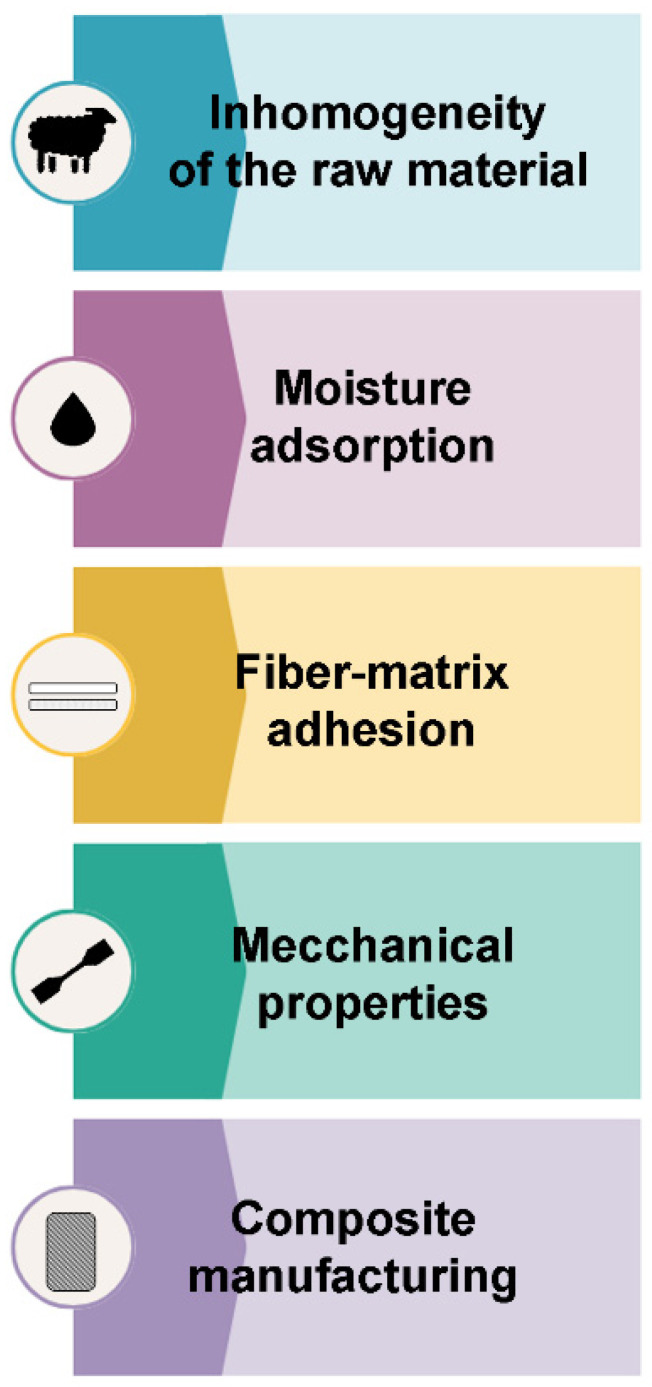
Challenges in wool-based composite.

Despite the promising potential of wool fibers as reinforcement in composites, several drawbacks and limitations remain, which could present challenges in large-scale production. A brief overview is presented in Figure 17.

One of the primary limitations associated with the use of wool fibers is a common challenge faced by all-natural fibers, namely the inherent inhomogeneity of the raw material. The properties of wool fibers are inextricably linked to the geographical region in which the sheep are bred, and the harvesting method employed. Furthermore, the processing technique can also influence the properties of the fibers. In addition to the variability of fibers as feedstocks, the typical properties of wool fibers can also present challenges in the composition of the composite. Indeed, wool fibers are capable of absorbing a certain amount of moisture, which affects the stability of the composite materials. This makes the production and storage of the wool fiber-based composite challenging. Furthermore, the adhesion between the fiber and the matrix represents another significant challenge to the exploitation of wool fiber in composite material production. Nevertheless, further research is required to elucidate the mechanisms of fiber-matrix interactions. This presents a challenge to the optimization of composite performance and the design of new materials with enhanced properties. These shortcomings can impede the exploitation of wool fibers as reinforcement, as inadequate mechanical properties may be obtained in some instances [49]. However, these limitations and drawbacks can be overcome by the use of physical and/or chemical pre-treatment of the fiber surface, such as alkaline treatments and treatment with silanes or other chemical products. These techniques are capable of reducing the hydrophilicity of wool fibers, thereby improving the compatibility between the matrix and the fibers and increasing the interfacial bonding [68].

A variety of methodological approaches can be used to promote the development of composite technologies based on wool fibers. Prior to any further processing, it is of the utmost importance to develop efficient and reproducible methods to pre-treat wool fibers. This is necessary in order to obtain high-quality and homogeneous fibers. Subsequently, it is imperative to develop suitable surface treatments to enhance the adhesion between fibers and the various matrices, thereby facilitating the transfer of load and mechanical resilience. Moreover, efforts should be made to optimize the formulations of the matrix, taking into account the specific properties of wool fibers, in order to increase the performance of the composite. Another promising approach for future exploration is the hybridization of wool fibers with synthetic fibers, such as those derived from carbon or glass, with the aim of reducing the water absorption behavior and enhancing the overall mechanical properties of composite materials. Furthermore, a more comprehensive study on the sustainability of the process is warranted for the wool fiber composite. Indeed, comprehensive life cycle assessments (LCAs) of wool fiber-reinforced composites are scarce. The implementation of LCAs can assist in the quantification of the environmental benefits and impacts associated with the utilization of wool fibers in composite materials, thereby facilitating the selection of sustainable materials and the design of sustainable products. However, the cost-effectiveness and scalability of the process must also be taken into account. Further investigation is required to ascertain the economic viability of large-scale production of wool fiber composites. It is essential that studies concentrate on the development of cost-effective processing methods and the scalability of production techniques in order to guarantee their industrial applicability. Ultimately, the creation of a standardized testing protocol represents a significant research gap. The absence of standardized testing protocols for wool fiber composites impedes the comparability and reproducibility of research findings. The establishment of industry-wide standards for the testing and evaluation of these materials could facilitate broader adoption and innovation.

## 8. Conclusions

This review underscores the importance of waste management strategies to convert discarded materials into valuable resources, in line with European Union guidelines. The research demonstrates that waste wool fibers, typically deemed unsuitable for textiles, can be effectively utilized as fillers in polymer composites, offering potential benefits for various applications, such as biomedical and construction sectors. Despite some challenges in optimizing wool fiber composites, the findings confirm the potential of waste wool fibers to significantly enhance the properties of composites, contributing to environmental sustainability. By integrating waste wool fibers into composite materials, this approach not only reduces waste but also fosters the development of innovative, sustainable materials that can be adopted across diverse industries. Future research should focus on refining processing techniques and exploring new applications to fully leverage the advantages of wool fiber composites.

## Figures and Tables

**Table 1:** Type of wool fibers.

Classification	Description	Reference
Fine wool	The fineness of a wool fiber can be used to determine its quality and price. In the international market, wool is typically described as fine when the fibers have a length greater than 65 mm and fineness within a range of 15 to 25 microns. This type of wool can be used to develop luxury fabrics. Fine wool is derived from pure and indigenous breeds, such as merino wool. Nevertheless, crossbred varieties can also be employed to enhance fineness. Morphologic differences are observed between wool fibers classified as fine and other wool fibers. Fine wool exhibits longer fibers, a unique cuticle scale pattern, and typically possesses an ortho- and para-cortical cell structure that is responsible for the natural crimp observed in this fiber type.	[6]
Carpet wool	Wool fibers with an average fineness ranging from 30 to 50 µm and a minimum fiber length of 35 mm are generally classified as carpet wool. These can be derived from either crossbred wool, including Lincoln, Romney, Drysdale, and Elliotdale, or wool produced in semi-arid areas. This category encompasses wool fibers with a thickness of 25–31 microns, derived from the crossbreeding of fine wool sheep with local breeds, such as Dorset, Southdown, and Suffolk. In the case of carpet wool, the ortho-cortical cell is surrounded by the para-cortical cell, resulting in a reduction in crimp and a corresponding increase in marginal mechanical properties.	[7,8]
Coarse wool	Wool fiber with a fineness of 50–80 μm and 40–80% hairy-type continuous medullation is regarded as coarse wool. This wool can be derived from sheep of the arid and semi-arid areas. In comparison to fine and carpet wools, coarse wool exhibits a reduced proportion of ortho-cortical cells relative to para-cortical cells, which results in a compromised tensile strength. Moreover, the majority of coarse wool and hair are naturally pigmented	[9]
Kemp wool	Wool fiber with a fineness of more than 60 μm and a continuous medullation of more than 80% is considered kemp wool. The wool in question can be derived primarily from the mutton sheep breed, which is generally considered to have a lower economic value due to its poor quality. It is brittle in nature due to the absence of crimp, chalky white in color, and exhibits a lack of exhaustion when dyed. Additionally, it is relatively weak. They are employed principally as a constituent of blended products with coarse wool for technical textiles and floor coverings. In kemp wool, the proportion of cortical cells to the entire fiber is less than 20%.	[7,10]

**Table 2:** A comparative analysis of the properties of wool fiber composites with those of commonly used materials.

Material	Thermal Conductivity (W/m K)	Sound Adsorption Coefficient (α)	Advantages	Disadvantages	References
**Wool**	0.04–0.06	0.70–0.90	Renewable, biodegradable, excellent insulation properties	Sensitive to moisture, may require treatment for durability	[16,33]
**Rock wool**	0.03–0.04	0.90–0.95	High insulation efficiency, fire-resistant	Non-biodegradable, can cause skin irritation	[34,35]
**Polyurethane**	0.02–0.03	0.50–0.70	Lightweight, excellent thermal insulation	Non-renewable, emits toxic gases when burned	[36,37]
**Hemp Fiber composite**	0.04–0.06	0.60–0.80	Renewable, biodegradable, good insulation properties	Variable quality, sensitive to moisture	[38,39]
**Cellulose insulation**	0.04–0.045	0.70–0.85	Recycled material, good thermal and acoustic insulation	Can settle over time, sensitive to moisture	[40,41]
